# Work-related musculoskeletal disorders in the automotive industry due to repetitive work - implications for rehabilitation

**DOI:** 10.1186/1745-6673-5-6

**Published:** 2010-04-07

**Authors:** Michael Spallek, Walter Kuhn, Stefanie Uibel, Anke van Mark, David Quarcoo

**Affiliations:** 1 Institute of Occupational Medicine, Charité-Universitätsmedizin Berlin, Free University Berlin, Humboldt-University Berlin, Thielallee 69-73, 14195 Berlin, Germany; 2 Institute of Occupational Medicine, University Lubeck, Ratzeburger Allee 160, 23538 Lübeck, Germany

## Abstract

**Background:**

Musculoskeletal disorders (MSDs) due to repetitive work are common in manufacturing industries, such as the automotive industry. However, it's still unclear which MSDs of the upper limb are to be expected in the automotive industry in a first aid unit as well as in occupational precaution examinations. It is also unclear which examination method could be performed effectively for practical reasons and under rehabilitation aspects. Additionally, it was to discuss whether the conception of unspecific description for MSDs has advantages or disadvantages in contrast to a precise medical diagnosis.

**Methods:**

We investigated the health status of two study populations working at two automotive plants in Germany. The first part included 67 consecutive patients who were seen for acute or chronic MSDs at the forearm over a 4-month period at the plants' medical services. Information about patients' working conditions and musculoskeletal symptoms was obtained during a standardized interview, which was followed by a standardized orthopedic-chiropractic physical examination. In the second part, 209 workers with daily exposure to video display terminals (VDT) completed a standardized questionnaire and were examined with function-oriented muscular tests on the occasion of their routine occupational precaution medical check-up.

**Results:**

The majority of the 67 patients seen by the company's medical services were blue-collar works from the assembly lines and trainees rather than white-collar workers from offices. Rates of musculoskeletal complaints were disproportionately higher among experienced people performing new tasks and younger trainees. The most common MSD in this group were disorders of flexor tendons of the forearm. By contrast, among the 209 employees working at VDT disorders of the neck and shoulders were more common than discomfort in the forearm. A positive tendency between restricted rotation of the cervical vertebrae and years worked at VDT was observed. In addition, only less than 8% of unspecific disorders of the upper limb (esp. wrist and forearm) were found.

**Conclusions:**

Functional tests for the upper limb seemed to be very helpful to give precise medical advice to the employees to prevent individual complaints. The results are also helpful for developing specific training programs before beginning new tasks as well as for rehabilitation reasons. There's no need to use uncertain terminology (such as RSI) as it may not be representative of the actual underlying disorders as diagnosed by more thorough physical examinations.

## Introduction

The increased automation of the car manufacturing process, in which much of the assembly has been delegated from man to machine, has done much to relieve workers the burden of heavy lifting [1]. However, despite ergonomic improvements in the workplace, many jobs still require workers to perform repetitive tasks [2,3]. Investigations into the health aspects of repetitive work have demonstrated some years ago the prevalence of unspecific diagnosis (for example "repetitive strain injuries" RSI) has been increasing in different countries around the globe [4,5]. Other sectors of the workforce, especially those working in offices at video display terminals (VDTs), have also been reported to suffer from work-related problems [6]. VDT-work, such as data-entry, can cause disorders of the wrist and forearm, especially in poorly designed workspaces [7-10].

The main objective of this two-part study was to determine the type of upper limb musculoskeletal disorders (MSDs) among both blue-collar (assembly line, trainees) and white-collar (office) workers in large automotive manufacturing plants. A secondary objective was to relate the individual disorders to the results of the functional examinations and, if possible, to type of repetitive work they perform. It was also of interest whether functional testing can be helpful in developing individual training or rehabilitation programs. Additionally, it was of interest whether the conception of unspecific descriptions for MSDs in the upper extremity has advantages compared with functional-oriented muscular tests and developing a precise medical diagnosis.

## Participants and Methods

### Participating Institutions

Workers from two automotive manufacturing plants in Germany participated in this study. Plant A assembled gearboxes and small service parts for two German car manufacturers, plant B manufactured light duty vehicles and small passenger busses. Both plants employed roughly 15,000 workers each, only a quarter of whom were white-collar workers. Trainees comprised approximately 5% of the workforce in both plants. Blue-collar work included repetitive tasks such as assembling product, connecting cables, polishing metal and drilling (Plant A) respectively working in a press shop, body shop or car assembly lines (Plant B). All participants provided informed consent.

### Data collection

The study group in the first part of the investigation consisted of 67 consecutive employees who were seen on their own decision in the company's medical service units in plant A over the 4-month study period for acute or chronic musculoskeletal disorders in the forearm (42 male: age: 38,5+/- 10 y, height 176,7 cm +/- 7,0 cm, weight: 78,7 kg +/- 10,2 kg, 25 female: age 27,1 +/- 14,2 y, height 165,0 cm +/- 6,9 cm, weight 61,8 kg +/- 11,8 kg). Seventy-four percent of the participants were blue-collar workers, whose jobs required manual force; only 16% of the participants worked at computer monitors for more than 6 hours per day. Information about the presenting symptoms and the patient's respective working environment were obtained during a standardized interview, after which an experienced occupational physician examined the patient. The physical examination of affected joint and other selected joints consisted of range of motion tests and resisted motion maneuvers (table 1). Range of motion data were recorded as described by Rompe and Erlenkämper and Kapandji [11,12]. If no plausible correlation could be found between the worker's MSD at presentation and the type of work he or she performed or at invidual information on other specific loads, the occupational physician visited the workplace to find possible ergonomic stressors. Visitation of the workplace was necessary in less than ten percent of the cases.

**Table 1:** Orthopedic-Chiropractic Diagnostic Method for Disorders of the Forearm [18]

Elbow/Forearm/Wrist
Pronation/Supination (with and without resistance)

Extension/Flexion (with and without resistance)

Ulnar-/Radialduction (with resistance)

Abduction and adduction of the fingers (with resistance)

Extension and adduction of the thumb (with resistance)

The second cohort in Plant B consisted of 209 white-collar workers who worked at video display terminals. In addition to a regular occupational medical check-up examination for employees at VDU called G 37 [13], each participant completed a questionnaire developed by Läubli et al. [14] to obtain demographical data, work history, current working conditions with VDT-exposure data, current musculoskeletal complaints and relevant medical history. Each participant submitted also to a visual acuity test and was asked to participate in an extended orthopedic-chiropractic examination of the cervical vertebrae and upper limbs (table 2). The participation rate for the functional examination was 97,2%. Although all participants worked at video display terminals at the time of assessment, an index of daily VDT-exposure (in hours) multiplied by the years of VDT-work was calculated to estimate the total level of VDT-exposure to prove a dose-response-relationship between VDU work and MSDs. An employee received the highest index (> 40), if he or she had spent more than 10 years working more than half of each shift at a video display terminal. The participants spent an average of 3.3 hours/day (± 2.2 hours/day) working at a VDT and had on average 5.5 years (± 3.9 years) of VDT-work experience. Age, height and weight were normally distributed within the study group.

**Table 2:** Orthopedic-Chiropractic Diagnostic Method for Disorders of the Upper Limb, Shoulder and Neck [18]

Elbow/Forearm/Wrist
Pronation/Supination (with and without resistance)

Extension/Flexion (with and without resistance)

Ulnar-/Radialduction (with resistance)

Abduction and adduction of the fingers (with resistance)

Extension and adduction of the thumb (with resistance)

**Cervical Spine**

Rotation in normal position (neutral)

Rotation in ante-/retroflexion

Side-flexion

Extension/Flexion

Neck-compression/neck-traction

**Shoulder**

Elevation (painful arc yes/no)

Inner-/Outer-rotation (with and without resistance)

Flexion/Extension of the elbow (with resistance)

Adduction/Abduction (with resistance)

## Results

**Figure 1:**
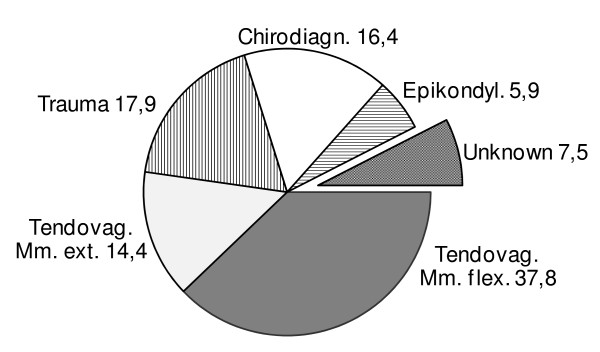
**Diagnosis rates [%]**.

Of the 67 workers in the first part of the study, the majority (52.5%) were diagnosed with clinical signs for tendovaginitis of the forearm. Disorders of flexors of the forearm outnumbered disorders of the forearm extensors almost 3:1. The diagnosis rates of traumatic injuries and treatable chiropractic disorders were roughly equivalent (fig 1). Due to the repetitive nature of the manual work in the assembly lines, requiring twisting and pinching motions, the rates of epicondylagia among the employees in this investigation were in the same range as described in other occupational health studies [15]. The functional tests performed during the physical examination led to a precise diagnosis of the underlying MSD in 92.5% of the cases without the need for imaging. The diagnosis was unclear only in 7,5% of the cases, but none of the participants in the study group was diagnosed with unspecific diagnosis terms like RSI. Furthermore, the results of the functional tests informed the physician's decisions about treatment options and employee sick leave as well as on the need for effective preventive training or rehabilitation aspects.

Occupational analysis of the participating employees yielded the following results: white-collar workers were less affected by MSDs of the upper extremity relative to blue-collar workers and is in line with results described by Huisstede et al [16]. The highest rates of such musculoskeletal disorders were found in trainees and in more experienced blue-collar workers, who had recently been subject changes in either job tasks or the normal sequence of task execution. Thus, before companies change the type or sequence of tasks performed by assembly line workers, they should invest in employee training and rehabilitation programs to optimize the ergonomic environment and prevent workers' exposure to unnecessary occupational health hazards.

**Figure 2:**
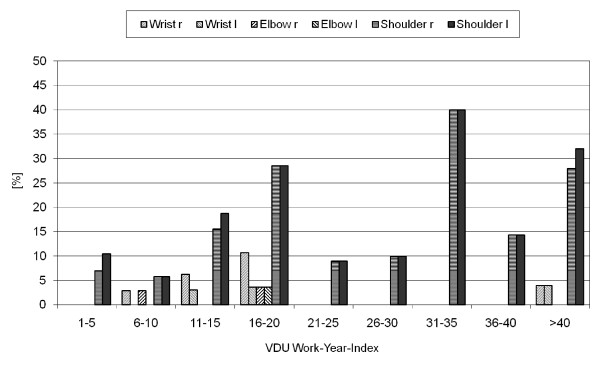
**Complaints in the upper extremity vs Index, n = 209**.

**Figure 3:**
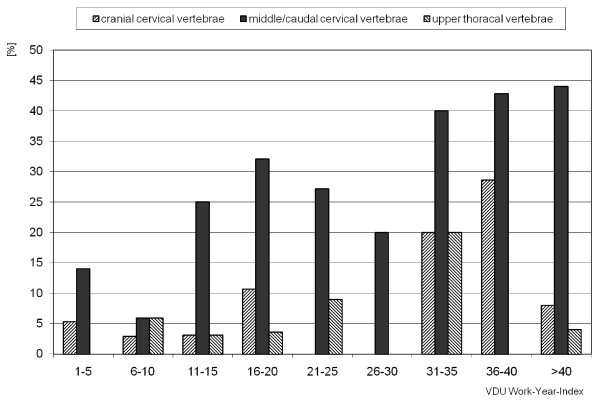
**Disorders of the cervical/thoracical vertebrae vs. Index, n = 209**.

Although white-collar workers in the first part of the study were less affected by acute (or chronic) MSDs of the upper extremity, the study of the 209 employees working at VDTs demonstrated that 24.4% of the subjects had restrictions of motion. The shoulder region and the neck were more often affected than the forearm (fig 2). Ninety-seven percent of the subjects had neither remarkable functional test results nor restrictions of movement of wrist and forearm. Functional tests of movement in the neck, shoulder and arm showed the greatest reductions in the rotation and side-flexion of the middle and lower cervical vertebrae. Although the functional tests correlated with VDT-work and the type of injury, statistical analysis of the disorders of the middle cervical vertebrae and upper thoracic vertebrae showed only a positive tendency with increasing VDT-work experience index (fig 3)

## Discussion

In the two studies we were able to demonstrate the usefulness of systematic functional tests in the diagnosis of work-related musculoskeletal disorders of the upper limb of workers due to long-term occupational stress in the automotive industry. The disorders of the forearm flexor tendons were most common among blue-collar workers at assembly lines and trainees, whereas the cervical rotation and side-flexion was most affected in white-collar workers with different VDT tasks, which is in good accordance with information in the literature [17]. Despite the high reliability of the functional diagnostic examination of the affected joints [18,19], no chronic musculoskeletal disorders were found in either study group with respect to the occupational stress over years of employment.

Controversy remains in occupational health literature as to the prevalence of unspecific diagnosis terms like RSI in industries with assembly line and VDT-work [20]. The results of the functional physical examination tests in our study substantiated in more than 92% of the patients a precise medical diagnosis. There was no need to use unspecific descriptions like RSI and our results are good in line with the recommendations published by Sluiter et al [21]. The value of terms such as RSI or occupational overuse syndrome (OOS) has been called into question by those who argue that these terms describe symptoms rather than distinct medical conditions [3,4]. The overwhelming majority of the MSDs in our study could be diagnosed according to the physical and functional examination and an assessment of the subject's medical history and ergonomic stressors of the work environment. We agree with van Tulder's opinion, that RSI or OOS are not a correct diagnosis, but only an umbrella term like cumulative trauma disorders, work related upper limb disorders etc. [3].

In this study, methodological functional testing allowed the occupational physician to identify and specifically diagnose injured structures as described by Kuhn and Spallek [22]. The results also emphasize the importance of individual training at the workplace as well as preventive ergonomic actions, individual gym exercises and rehabilitation programs [23]. With respect to our results, we recommend that more rigorous criteria for the physical examination and diagnosis of work-related musculoskeletal disorders should be devised to support ergonomic changes in the workplace and to avoid equivocal diagnoses in the future.

## Competing interests

The authors declare that they have no competing interests.

## Authors' contributions

MS and WK carried out the physical examinations and drafted the manuscript, SU and AvM participated in the design of the study and in the evaluation. DQ contributed to the discussion and interpretation of the results. All authors read and approved the manuscript. Part of this study was presented at the XIIIth Annual International Occupational Ergonomics and Safety Conference.
